# The Influence of *Helicobacter pylori* on Human Gastric and Gut Microbiota

**DOI:** 10.3390/antibiotics12040765

**Published:** 2023-04-16

**Authors:** Marcello Fiorani, Ege Tohumcu, Livio Enrico Del Vecchio, Serena Porcari, Giovanni Cammarota, Antonio Gasbarrini, Gianluca Ianiro

**Affiliations:** 1 Digestive Disease Center, Fondazione Policlinico Universitario Agostino Gemelli IRCCS, 00168 Rome, Italy; 2 Dipartimento Universitario di Medicina e Chirurgia Traslazionale, Università Cattolica del Sacro Cuore, 00168 Rome, Italy

**Keywords:** gastric microbiota, gut microbiota, *Helicobacter pylori*, probiotics

## Abstract

*Helicobacter pylori* is a Gram-negative bacterium that is able to colonize the human stomach, whose high prevalence has a major impact on human health, due to its association with several gastric and extra-gastric disorders, including gastric cancer. The gastric microenvironment is deeply affected by *H. pylori* colonization, with consequent effects on the gastrointestinal microbiota, exerted via the regulation of various factors, including gastric acidity, host immune responses, antimicrobial peptides, and virulence factors. The eradication therapy required to treat *H. pylori* infection can also have detrimental consequences for the gut microbiota, leading to a decreased alpha diversity. Notably, therapy regimens integrated with probiotics have been shown to reduce the negative effects of antibiotic therapy on the gut microbiota. These eradication therapies combined with probiotics have also higher rates of eradication, when compared to standard treatments, and are associated with reduced side effects, improving the patient’s compliance. In light of the deep impact of gut microbiota alterations on human health, the present article aims to provide an overview of the complex interaction between *H. pylori* and the gastrointestinal microbiota, focusing also on the consequences of eradication therapies and the effects of probiotic supplementation.

## 1. Introduction

### 1.1. Helicobacter pylori

*Helicobacter pylori* (*H. pylori*) is a Gram-negative, microaerophilic, spiral bacterium with a diameter of approximately 3 μm, which is able to colonize the human stomach. More than half of the world’s population is estimated to be infected, with approximately 4.4 billion *H. pylori*-positive individuals worldwide in 2015. The global prevalence is higher in adult males and in subjects living in developing countries, with a wide range of variability, from 24% in Oceania to 70% in Africa [1,2,3].

The high prevalence of *H. pylori* has a huge impact on human health, as it has been associated with several gastric and extra-gastric disorders [4,5]. *H. pylori* infection is one of the most common causes of gastritis and peptic ulcer [6], as well as gastric cancer [7]. Nearly 2–3% of patients with *H. pylori* can develop gastric adenocarcinoma, and 0.1% will experience mucosa-associated lymphoid tissue (MALT) lymphoma [8]. However, *H. pylori* has also been negatively associated with several extra-gastric digestive disorders, including, coeliac disease, inflammatory bowel disease (IBD), Barrett’s esophagus, and gastroesophageal reflux disease (GERD) [5]. Notably, the pathogenesis of the gastrointestinal disorders associated with *H. Pylori* could also be influenced by the gastric microbiota. Interestingly, it has been shown that the presence of non-*H. pylori* bacteria in the gastric microbiota can influence the development of gastric cancer, even after H. pylori eradication, but their role is still not well defined [9].

*H. pylori* has developed several strategies to increase its survival within the human stomach, through a complex interplay with the gastric ecosystem and the immune system. 

One of the main mechanisms that mediate its survival and persistence within the gastric environment is the production of urease [10,11]. Through the action of Urel, a pH-gated urea channel that allows urea entry in an acid environment, intrabacterial urease activity is activated. Urea is thus hydrolyzed, with the production of ammonia (NH_3_) and CO_2_, consequently alkalizing the gastric pH [12,13]. NH_3_, which can disrupt tight cell junctions and the gastric epithelium, and CO_2_, which can interfere with host bactericidal strategies, are involved in the pathogenesis of complications by *H. pylori* infection [14]. Moreover, H. pylori produces specific virulence factors, including the vacuolating cytotoxin A (VacA), the cytotoxin-associated gene A (CagA) product, sialic acid-binding adhesin A (SabA), and the high-temperature requirement A (HtrA) protease [15]. VacA leads to the vacuolation and apoptosis of the host cell, disrupting membrane transport proteins, mitochondrial activities, endocytic trafficking, and uncontrolled activation of MAP kinases [16]. VacA toxin also inhibits the degradation of the epidermal growth factor (EGF) and increases the activity of T regulatory cells, reducing the host immune response to *H. pylori* and easing its persistence in the gastric environment [17]. CagA toxin is an oncoprotein with cytotoxic and immunosuppressant activities [18], associated also with increased bacterial motility [19], impairing specifically cytoskeleton activities and the MAP kinase pathway [15]. In a recent meta-analysis, CagA was the most common risk factor for non-cardial gastric cancer (OR = 3.22; 95%CI: 2.10–4.94), while patients infected with VacA-positive strains were associated with a doubled neoplastic risk (OR = 2.05; 95%CI: 1.67–2.52) [20].

HtrA, an E-cadherin protease of the extracellular compartment, damages the adherent junctions and the epithelial barrier, favoring *H. pylori* invasion through the gastric epithelium [21]. Therefore, specific HtrA polymorphisms have been directly linked to an increased risk of gastric cancer [22].

Other pathogenic pathways are the production and expression of nearly 64 specific outer membrane proteins (OMPs) [10], including blood group antigen-binding adhesin A (BabA), sialic acid-binding adhesin A (SabA) [23], HopQ [24], Helicobacter outer membrane B (HomB), and outer inflammatory protein A (OipA) [25]. In particular, BabA and SabA are fundamental in the adhesion process, while HopQ plays an essential role as membrane porin to transport toxins into the host cell, and HomB and OipA act as inhibitors of the apoptotic cascade and as pro-inflammatory factors. Therefore, OMPs such as HomB or blood group antigen-binding adhesion 2 (BabA2) are more expressed by the strains found in the presence of peptic ulcer (OR = 1.36; 95%CI: 1.07–1.72) and gastric cancer (respectively, OR = 2.16; 95%CI: 1.37–3.40; and OR = 2.05, 95%CI: 1.30–3.24; *p* = 0.002) [26,27].

Other relevant strategies employed by *H. pylori* to improve its survival within the stomach include the release of outer membrane vesicles (OMVs), which contain various molecules such as LPS, peptidoglycan, outer membrane proteins, and virulence factors. These vesicles mediate the communication between the host environment and the bacteria and can be employed to introduce *H. pylori* products into host cells, where they can promote infection, regulate immune responses and impair cellular functions [28]. OMVs could also be involved in bacterial mechanisms of antibiotic resistance [29].

Moreover, *H. pylori* is able to switch from a spiral, vegetative form to a coccoid one in case of adverse conditions, e.g., the poor availability of nutrients, increasing the chance of long-lasting colonization [30]. This is due to not only its higher resistance to antibiotics but also because the coccoid form can be hardly cultured and often results in false-negative diagnoses [31].

Due to its association with various disorders, including gastric cancer, *H. pylori* infection requires adequate intervention upon diagnosis [32], and a series of antibiotic regimens have been trialed to eradicate it. Multiple classes of drugs are used in combination, as triple or quadruple therapies [33]. The principal components of these therapies are microbial agents, such as imidazole (metronidazole or tinidazole), macrolides (clarithromycin or azithromycin), tetracycline, amoxicillin, rifabutin, and furazolidone [34,35]. Regardless of the potential antibiotic resistance that may be present, the use of bismuth components in quadruple therapy increases the rates of eradication [36]. In combination with antimicrobial agents, proton-pump inhibitors (PPIs) contribute to the treatment process by decreasing the acid secretion in the stomach, allowing previously formed ulcerations to heal [37]. This blocking effect on the secretory mechanism is accomplished through the inhibition of the H+/K+ adenosine triphosphatase (ATPase) enzyme on the parietal cell canalicular membrane [38]. Other than attenuating the acidity of the gastric environment, PPIs also help by enhancing the stability of acid-labile antibiotics, increasing the gastric pH [37].

First-line therapies should be chosen depending on the local prevalence of clarithromycin resistance, and a triple regimen is recommended in regions with a low prevalence of clarithromycin resistance. This combination includes a standard dose of PPI, amoxicillin (1 g), and clarithromycin (500 mg) twice daily for 7–14 days. A quadruple therapy containing bismuth can be implemented as a first-line therapy in regions with high (>15%) or unknown prevalence of clarithromycin resistance [33]. The rising trend of resistance to first-line therapies has put forward the concept of susceptibility testing pre-treatment, favoring a culture-guided approach [39]. Although susceptibility can be examined using both invasive and non-invasive approaches, upper endoscopy with specimen cultures and minimum inhibitory concentration calculation is considered the most common one [39]. However, given the challenge of standardizing the quality of the method and the scarce availability of expertise, this approach still has to be prioritized in the future [39].

### 1.2. The Human Microbiota

The gastrointestinal tract hosts trillions of microorganisms over its entire length, including bacteria, viruses, fungi, and protozoa [40,41,42], and the collection of their genetic material is called microbiota. The gut microbiota is a highly dynamic ecosystem and is influenced by multiple factors, such as diet, lifestyle, antibiotic therapies, long-term proton PPI consumption, and *H. pylori* infection [43,44]. The interaction between the host and microbiota plays a pivotal role when it comes to balancing health and disease states [45], as microbiota is involved in the regulation of several immune and metabolic pathways [46,47]. Indeed, a large body of evidence shows that microbiota alterations are associated with a wide spectrum of disorders, including gastrointestinal and liver diseases, allergies, cardiometabolic and autoimmune diseases, neurological disorders, and malignancies [48,49,50].

Bacterial-derived metabolites are important mediators in this interaction between the host and microbiota, and specific metabolites, such as short-chain fatty acids (SCFAs), play a key role in maintaining the host’s health [43]. The decreased abundance of SCFA-producing bacteria, such as *Faecalibacterium* and *Roseburia*, has been linked to a higher risk of type-1 and type-2 diabetes, liver cirrhosis, IBD, and atherosclerosis [51,52,53,54,55,56].

A crucial role in the maintenance of gut health is played by the mucus layer, which is present throughout the whole length of the gastrointestinal tract. The mucus layer activity is also regulated by the microbiota [57], and the expression of Muc2, the main glycoprotein of the mucus layer of the colon, has been positively associated with a higher abundance of pathogens and worse intestinal damage in mouse models [58]. Muc2 glycosylation is involved in safeguarding the protein structure against host and bacterial proteases, also facilitating the retention of water for the creation of a mucus gel. Microbial colonization has been shown to influence the glycosylation process of Muc2 [59].

The abovementioned aspects of the interaction between the host and the gut microbiota have also an important role during the *H. pylori* infection because this bacterium is not only located in the epithelium but also in the gastric surface mucous gel layer [60], and bacterial-derived metabolites such as SCFAs could inhibit its growth [61]. Thus, considering the profound effects of the gut microbiota on human health and the relevant role of *H. pylori* in influencing the host’s microbiota, the present review aims to offer a summary of the intricate interplay between *H. pylori* and the gastrointestinal microbiota, with a particular emphasis on the effects of eradication therapies and the impact of probiotic supplementation.

## 2. The Effect of *H. pylori* on Gastric Microbiota

Although the human stomach was previously considered unfit for bacterial growth, it has now been demonstrated that it is colonized by a complex microbial community [62]. The gastric microbiota is characterized by a reduced richness and diversity compared with the ileal and colonic microbiota, and it hosts fewer aero-intolerant species [63]. The most relevant phyla observed are Proteobacteria, Firmicutes, Bacteroidetes, Actinobacteria, and Fusobacteria, while the most important genera reported are Helicobacter, Streptococcus, Prevotella, Neisseria, Veillonella, Fusobacterium, and Haemophilus [9,64,65]. Furthermore, the gastric microbiota found in gastric biopsies is different from that observed in gastric juice, showing the presence of a distinct mucosal microbiota in the stomach [66].

The relationship between *H. pylori* and gastric microbiota could be mediated through multiple mechanisms, such as virulence factors, the modification of gastric acidity, host immune responses, and competition [67,68,69].

One of the main virulence factors of *H. pylori*, the cytotoxin-associated gene A (CagA), is capable of impairing cellular proliferation, apoptosis, cell motility, inflammatory response, and the arrangement of the cytoskeleton in host cells [15] and may also lead to gastric dysbiosis. In a transgenic Drosophila model of CagA expression, without the presence of *H. pylori*, Jones et al. observed that the virulence protein altered the host microbiota. Moreover, they found the increased activation of innate immunity to be a consequence of CagA-induced dysbiosis [70]. Furthermore, Wang et al. showed that in the gastric biopsies of patients affected by chronic gastritis or gastric cancer, the presence of different CagA and VacA virulence genotypes influenced the gastric microbiome’s composition [71].

The colonization of human gastric mucosa by *H. pylori* affects gastric luminal acidity, altering the expression of proton pumps and allowing the colonization of the gastric microenvironment to other microorganisms that are otherwise incapable of surviving in the stomach [72]. The acute phase of the infection results in an initial reduction in gastric pH, stimulating gastrin release, and the consequent abundance of *H. pylori* that predominates the microenvironment. On the other hand, chronic infection, leading to the development of atrophic gastritis, is associated with a decreased luminal acidity that favors the colonization of other bacteria, leading to microbial growth that can outnumber *H. pylori* [73].

The chronic *H. pylori* infection can progress over time through different stages according to the Correa cascade: chronic gastritis, atrophy, intestinal metaplasia, dysplasia, and eventually gastric adenocarcinoma. However, carcinogenesis could also progress independently from *H. pylori* or even after its eradication, suggesting the presence of other relevant factors in the gastric microenvironment participating in the process [9,74]. Moreover, even though multiple studies have shown that the gastric microbiota is altered in various gastric diseases, they have revealed heterogeneous results, without identifying the specific microbial signatures associated with different gastric pathologies [9].

The complex immune response to *H. pylori* is changeable during life. During infancy, this is mainly a regulatory inflammatory pattern, with higher concentrations of IL-10 and TGF-β1 with an increased number of mucosal FOXP3 + Treg cells. The predominantly regulatory pattern allows the gastric environment to be more vulnerable to *H. pylori*, but there is a mild degree of inflammation and mucosal damage. On the other hand, in adults, there is a predominant Th1 response, with higher mucosal levels of IFN-γ and IL12p70 [75]. Adults also have an increased Th17 response, with higher levels of IL-17A and IL-23 and lower concentrations of TGF-β1, with a cytokine profile more associated with epithelial damage [76]. Another mechanism involved in this crosstalk involves antimicrobial peptides such as defensins, molecules known to be active against bacteria, viruses, fungi, and protozoa, which are increased in *H. pylori*-induced gastritis [77]. Furthermore, *H. pylori* can produce cecropin-like antibacterial peptides that have an important bactericidal effect and proinflammatory activity [78].

Beyond these pathways, *H. pylori* is able to predominate over other gastric bacteria. When *H. pylori* colonizes the gastric mucosa, it succeeds in becoming the most abundant organism, accounting for 40–90% of the gastric microbiota composition [45,64,79]. Consequently, the presence of *H. pylori* leads to a significant decrease in gastric alpha diversity [79,80]. In general, in *H. pylori*-positive subjects, there is an increased abundance of Proteobacteria, likely due to the contribution of *H. pylori* itself, while the abundance of Actinobacteria, Bacteroidetes, Fusobacteria, and Firmicutes is reduced [79,81]. In a study by Wang et al., some species, such as *Stenotrophomonas maltophilia*, *Chryseobacterium unclassified*, *Pedobacter unclassified*, *Stenotrophomonas unclassified*, *Variovorax unclassified*, and *Pseudomonas stutzeri*, have been associated with the presence of *H. pylori* infection, through the shotgun sequencing of stomach swab samples of 96 patients [82]. On the other hand, in a rhesus macaque model, Martin et al. observed the impact of *H. pylori* on the antecedent gastric microbiota and found no significant variations in the relative abundance of non-Helicobacter taxa, indicating that in the rhesus macaque model, the gastric microbiome is stable despite the presence of the *H. pylori* infection [83].

**Figure 1:**
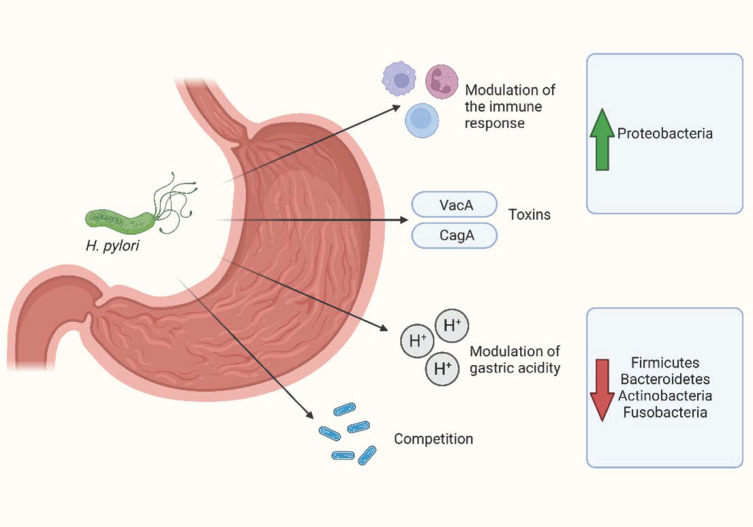
Main mechanisms mediating the relationship between *H. pylori* and gastric microbiota. Created with BioRender.com.

There is also initial evidence of the influence of *H. pylori* on the gastric microbiota of pediatric patients. In a cohort of 122 children with gastrointestinal symptoms, 57 of whom were diagnosed with *H. pylori* infection, Zheng et al. analyzed the gastric microbiome from mucosal biopsies [84]. The authors observed that the group of patients diagnosed with *H. pylori* infection (*H. pylori*-positive group) had a lower gastric bacterial diversity than those without *H. pylori* infection and that the two groups had a significantly different compositions based on beta diversity. In the *H. pylori*-positive group, the relative abundance of Actinobacteria, Bacteroidetes, Firmicutes, Fusobacteria, Gemmatimonadetes, and Verrucomicrobia was significantly decreased compared with the *H. pylori*-negative group. Moreover, at the genus level, Achromobacter, Devosia, Halomonas, Mycobacterium, Pseudomonas, Serratia, Sphingopyxis, and Stenotrophomonas were more abundant in the *H. pylori*-negative group, while only Helicobacter was more abundant in the *H. pylori*-positive group. The relative abundance of the Helicobacter genus was significantly different among the *H. pylori*-positive patients, with levels ranging from 2.19% to 80.98% [84]. Figure 1 illustrates the main mechanisms mediating the relationship between *H. pylori* and the gastric microbiota. 

## 3. The Influence of *H. pylori* on Gut Microbiota

The presence of *H. pylori* in the human organism can influence the composition of the gut microbiota through several pathways, including a direct effect of the infection or as a consequence of *H. pylori* eradication regimens.

Although *H. pylori* infection can have systemic effects and consequently influence not only the gastric microbiota but also the microbial communities of the whole gastrointestinal tract [45,85], relatively few studies have investigated the influence of the infection itself on the human gut microbiota.

In some studies, patients infected by *H. pylori* presented with increased diversity of gut microbiota compared with uninfected controls [86,87]. However, this association was not confirmed in other studies [64,88]. In an animal model of Mongolian gerbils, the *H. pylori* infection caused a distinct and prolonged shift in gut microbiota composition [89]. Moreover, in a large study of 212 patients with *H. pylori* and 212 matched controls [87], *H. pylori* infection was significantly associated with increased microbial diversity and fecal microbiota alterations, including a decreased abundance of Parasutterella and increased levels of Haemophilus and Pseudoflavonifractor in the infected cohort. Moreover, the *H. pylori* antigen load was correlated with larger alterations in the gut microbiota than sex or age, while it was negatively correlated with the abundance of Bacteroides, Fusicatenibacter, Alistipes, and Barnesiella [87].

Beyond its direct influence, the major impact of *H. pylori* on the intestinal microbiota is mediated by drug-based eradication regimens, including antibiotics and PPIs, which can lead to significant changes in the composition of the human gut microbiome.

As is widely known, the use of antibiotics has various harmful effects on the gut microbiota, such as a reduction in microbial diversity, alterations in its metabolic activity, and the selection of antibiotic-resistant microorganisms [90]. The impact of antibiotic therapies on the gut microbiota is influenced by their class, administration route, duration, dosage, pharmacodynamics, and pharmacokinetics, as well as host-related factors, such as baseline microbiota composition, age, and lifestyle [91].

Antibiotic use seems to play a role in the development of several disorders, including irritable bowel syndrome, inflammatory bowel disease, metabolic disorders, and liver disease. A clear example of antibiotic-driven dysbiosis is *C. difficile* infection, which frequently follows antibiotic therapies [91].

The use of PPIs has been investigated in the context of several gastrointestinal disorders. Regardless of the therapy regimen they are included in, PPIs have been shown to induce changes in the gut microbiome, favoring the development of enteric infections, including *C. difficile* infection [92]. In a study including more than 200 participants, the chronic use of PPIs demonstrated a decrease in gut microbiome diversity [93]. On the level of different bacterial populations, changes such as an increase in *Actinomycetales* in families Streptococcoceae and Micrococcoceae and species *Lactobacillus salivarius* were observed [93].

A large study previously conducted has demonstrated that the 7-day antibiotic treatment for eradication profoundly disturbs the oral and colonic microbiota, and alterations can be observed even a week after treatment, persisting for up to 4 years [94]. Chen et al. compared the composition of fecal microbiota after 14 days of quadruple eradication therapy with healthy individuals’ samples, and the alpha diversity was remarkably lower after treatment. This poor diversity lasted for 6 weeks post-treatment [95].

Considering changes at the phylum level, there were some transient alterations demonstrated 14 days after treatment initiation; specifically, there was a decrease in the relative abundance of Firmicutes, Bacteroidetes, Verrucomicrobia, and Lentisphareae, while an increase in Proteobacteria and Cyanobacteria was observed when compared to the baseline. A decrease in the relative abundance of Lachnospiraceae and Ruminococcaceae was also observed; however, it was only transient and disappeared after 2–3 months [95]. Another study conducted on a pediatric patient population who underwent bismuth quadruple therapy for 10 days, showed a statistically significant decrease in alpha diversity in the short term, up until 6 weeks post-treatment. In the same population, no long-term changes were noted, nor at the phylum level [96]. Contrastingly, He et al. reported a higher alpha diversity after eradication therapy in asymptomatic young adults [97]. Another meta-analysis focusing on changes in the taxon level illustrated a decrease in Actinobacteria in patients who have undergone eradication therapy when compared to baseline healthy individuals. Proteobacteria showed only a transient increase in the short term [98].

The eradication treatment also has an outcome regarding ghrelin levels, which is known as the “hunger hormone” and is predominantly secreted by the endocrine cells of the stomach and from the duodenum to a smaller extent. It is well known as one of the key regulators of appetite [99]. Regarding the ghrelin levels after triple or quadruple therapy for eradication, Martín-Núñez et al. observed a statistically significant decrease in fasting plasma ghrelin levels compared to the pre-treatment levels. This trend was also reflected in the gut microbiota profiles, where the abundance of Bacteroides *Bifidobacterium longum* and *Parabacteroides distasonis* predicted positive fasting ghrelin levels, while an increased abundance of Prevotellaceae, primarily its genus Prevotella, was linked to a lower amount of fasting ghrelin secretion in the stomach [100]. Nonetheless, the direct correlation and effects of *H. pylori* infection and its eradication treatment is still a topic of interest for further investigation.

There is well-established evidence that the integration of certain probiotics into *H. pylori* eradication regimens increases their success rates [101]. For this reason, the use of specific probiotic strains, including *Saccharomyces boulardii*, different strains of *Lactobacillus* spp., and *Bifidobacterium* spp., has been recommended by the latest Maastricht guidelines for the management of *H. pylori infection* [33,102].

Probiotics are defined as live microorganisms that, when administered in adequate amounts, confer a health benefit on the host [103]. The addition of probiotics to the *H. pylori* eradication therapy can be beneficial in improving both the eradication rates and decreasing the incidence of side effects often associated with the treatment, allowing better adherence to the therapy regimen [104,105]. The incidence of these adverse events, such as nausea, vomiting, or antibiotic-associated diarrhea, can occur in 5% to 30% of cases during the eradication therapy and could lead to the discontinuation of the treatment [106,107].

The mechanisms that mediate the effect of probiotics against *H. pylori* are still not clear and could involve the production of antimicrobial substances, competition, the reinforcement of mucosal barrier, and the modulation of the immune response [108,109,110,111]. Probiotics are known to increase the availability of lactic acid, SCFAs, hydrogen peroxide, and bacteriocin [112]. While SCFAs show an intense antibacterial activity, lactic acid also shares this effect, also inhibiting urease activity [112,113]. A study that focused on the bacteriocin secreted by multiple *L. bulgaricus* strains observed that it was able to inhibit *H. pylori* growth [114]. Recently, an acid-resistant strain, *L. johnsonii*, which was extracted from the gastric juice of healthy individuals, was able to antagonize *H. pylori* in both in vitro and mice models, indicating its potential therapeutical role [115].

In a meta-analysis of randomized controlled trials, involving 13 RCTs and 2306 patients, LÜ and colleagues [108] showed that probiotic supplementation improved the eradication rates by 11% and reduced the incidence of antibiotic-related side effects by 8% compared with the control group. Similar results have been confirmed in other meta-analyses [116,117].

Some studies have evaluated the benefits of specific strains, such as *Saccharomyces boulardii*; in a meta-analysis by Zhou et al., given in combination with the standard eradication treatment, it resulted in an improvement in *H. pylori* eradication rates and a significant reduction in the incidence of adverse events, compared with the control group [118]. Another meta-analysis of randomized controlled trials focused on Lactobacillus supplementation, finding that the *H. pylori* eradication rate was superior to that of the control group. In a subgroup analysis, the authors observed that in the *L. casei* and *L. reuteri* groups, the eradication rate was increased, but in the group of patients who received *Lactobacillus GG*, there was no improvement. Regarding the impact on the rates of side effects, no differences were observed between the supplementation group and the control group [119].

The application of advanced technologies for the sequencing of the microbial genome has allowed building initial evidence on the effect of probiotics on the gut microbiota during *H. pylori* eradication therapies. A study by Chen et al. [95] reported the effect of the eradication therapy with or without the supplementation of probiotics on the gut microbiota profile and alpha diversity. Patients were randomized into two groups: the first group received a bismuth-containing quadruple therapy without any addition of probiotics, while the second group received the same therapy with the supplementation of *Clostridium butyricum*, a butyrate-producing probiotic.

In both groups, alpha diversity decreased after treatment, and this reduction persisted for up to 6 weeks. The changes in alpha diversity were similar between both groups.

Regarding microbiota changes, at the phyla level, the short-term outcome (up to 14 days after therapy) revealed some alterations. Compared with baseline profiles, Firmicutes, Bacteroidetes, and Verrucomicrobia showed a decrease in abundance, while Proteobacteria and Cyanobacteria increased. The authors also observed some specific changes only among patients who underwent therapy with probiotic supplementation: compared with the baseline, Fusobacteria and Tenericutes decreased, and Actinobacteria increased.

Another study instead demonstrated an alleviation in gastrointestinal symptoms, as well as an increase in alpha diversity, after receiving a specific probiotic containing *S. boulardii* [120]. The same patients showed a higher abundance of Enterobacteriaceae, while a decrease in the abundance of Bacteroidetes and Clostridia was observed upon treatment completion [120]. The amelioration of symptoms resulting from the use of probiotics in patients undergoing *H. pylori* eradication could be potentially attributed to their beneficial effect on the gut microbiota, but further studies are needed to clarify this issue. Therefore, these emerging data suggest that the use of therapy regimens integrated with probiotics should be more widely adopted.

## 4. Future Insights

*H. pylori* infection affects the composition and structure of the gastrointestinal microbiota through the regulation of multiple factors such as gastric acidity, host immune responses, antimicrobial peptides, and virulence factors, playing a role in the development of gastric pathology and extra-gastrointestinal diseases. Some studies have also focused on the gastric microbiome beyond *H. pylori*, starting to clarify its impact on gastrointestinal health. However, most of the available studies have a retrospective and associative design, while future longitudinal and prospective studies may evaluate the dynamic process of the infection, hopefully also with a mechanistic approach, with the aim of clarifying the functional interaction between *H. pylori*, the gastric microbiome, and the host, and identifying novel biomarkers in order to prevent the development of gastric cancer.

Moreover, *H. pylori* eradication requires the administration of large doses of antibiotics and PPIs, but the impact of this treatment affects microbial communities through the entire gastrointestinal tract, with clinical effects that can last several weeks and have detrimental consequences for the gut microbiota, including a reduction in alpha diversity and beneficial taxa (e.g., Verrucomicrobia), and increased relative abundance of Proteobacteria [121,122]. Therapy regimens integrated with probiotics have shown higher rates of eradication than standard treatments, mainly because of a reduction in gastrointestinal symptoms and, consequently, higher compliance with therapies. Interestingly, probiotics may also reduce the impact of antibiotic treatment on the gastrointestinal microbiota, preserving its diversity. This issue, as well as the application of other therapeutic modulators of the gut microbiota to *H. pylori* eradication therapies, represent a valuable topic to expand upon in the upcoming years.

## Data Availability

Not applicable.
